# Intra-articular Injection of Kartogenin-Incorporated Thermogel Enhancing Osteoarthritis Treatment

**DOI:** 10.3389/fchem.2019.00677

**Published:** 2019-10-18

**Authors:** Shao-Jie Wang, Ji-Zheng Qin, Tong-En Zhang, Chun Xia

**Affiliations:** ^1^Department of Joint Surgery and Sports Medicine, Xiamen University Zhongshan Hospital, Xiamen, China; ^2^Medical School of Xiamen University, Xiamen, China

**Keywords:** chondrocytes, osteoarthritis, cartilage regeneration, kartogenin (KGN), thermogel

## Abstract

To provide a vehicle for sustained release of cartilage-protective agent for the potential application of osteoarthritis (OA) treatment, we developed a kartogenin (KGN)-incorporated thermogel for intra-articular injection. We fabricated a poly(lactide-co-glycolide)-block-poly(ethylene glycol)-block-poly(lactide-co-glycolide) (PLGA–PEG–PLGA) thermogel as a KGN carrier for IA injection. OA chondrocytes were cultured in thermogel with or with no KGN to investigate the effect of KGN thermogel on cartilage matrix. The *in vivo* effect of KGN thermogel on OA was examined in a rabbit OA model. The KGN thermogel showed a sustained *in vitro* release of KGN for 3 weeks. OA chondrocytes proliferated well both in thermogel and KGN thermogel. In addition, OA chondrocytes produced higher amount of [type 2 collagen (COL-2) and glycosaminoglycan (GAG)], as well as lower level of matrix metalloproteinase 13 (MMP-13) in KGN thermogel that those in thermogel with no addition of KGN. The gene analysis supported that KGN thermogel enhanced expression of hyaline-cartilage specific genes Col 2 and AGC, and inhibited the expression of MMP-13. Compared with intra-articular injection of saline or thermogel containing no KGN, KGN thermogel can enhance cartilage regeneration and inhibit joint inflammation of arthritic knees in a rabbit ACLT-induced OA model at 3 weeks after the injection. Therefore, the KGN-incorporated PLGA–PEG–PLGA thermogel may provide a novel treatment modality for OA treatment with IA injection.

## Introduction

Osteoarthritis (OA) is a common degenerative joint pathology affecting 151 million people worldwide. Direct intra-articular injections of drugs are commonly used to improve the joint bioavailability while minimizing systemic complications. Recently a small molecule, kartogenin (KGN), has been reported to promote collagen synthesis (Johnson, 2012). Intra-articular injection of KGN has been reported to enhance cartilage regeneration (Kang et al., 2014; Mohan et al., 2016; Fan et al., 2018). However, KGN cannot provide long-term therapeutic effects due to fast clearance and short retention of KGN in joints, posing a disadvantage in its clinical application. In order to improve residence time of treatment agents in joints, researchers have employed hydrogels for drug delivery and drug control release (Seliktar, 2012).

Poly(lactide-co-glycolide)–poly(ethylene glycol)-poly(lactide-co-glycolide) (PLGA–PEG–PLGA) triblock copolymer has been used as a potential matrix of thermogel, and can dissolve in water at low temperature (e.g., 4°C), and the solution gels around body temperature (i.e., 37°C) (Yu et al., 2011; Li et al., 2012; Wang et al., 2016c, 2019; Zhang et al., 2019). Given the advantages of possessing and minimally invasive way of delivering bioactive molecules, in the current study we used PLGA–PEG–PLGA copolymer to fabricate thermogel incorporated with therapeutic concentration of KGN for intra-articular injection. KGN thermogel system was evaluated both *in vitro* and *in vivo* to examine the potential for OA treatment. We cultured chondrocytes pre-treated with IL-1β to mimic OA chondrocytes (Cui et al., 2016) in to investigate the effect of KGN thermogel on OA chondrocytes in terms of cartilage matrix production and degradation. Then the PLGA–PEG–PLGA thermogel with or without KGN was injected in to OA knees in rabbits to examine the effects of KGN thermogel on OA.

## Materials and Methods

### PLGA–PEG–PLGA Thermogel Preparation

PLGA–PEG–PLGA triblock copolymers were purchased from (Daigang Co., Ltd., Jinan, Shandong, China). The copolymers were synthesized through the ring-opening polymerization (ROP) of L-LA and GA with PEG as a macroinitiator and Sn(Oct)2 as a catalyst as previously reported (Wang et al., 2016c). The Mns of PEG and PLGA were 1,500 and 1,400 g/mol, respectively. The molar ratio of L-LA and GA in PLGA segment is 75:25. PLGA–PEG–PLGA triblock copolymers was dissolved in PBS (pH 7.4) to obtain a 20 wt% gel solution, which was then kept at 4°C before being used in the following experiments. The sol—gel transition behavior of PLGA-PEG-PLGA thermogel was confirmed by incubation at 37°C for 15 min.

### Preparation of KGN Thermogel

Ten milligrams of KGN (Selleck Chemicals, Shanghai, China) was dissolved in 0.6 ml dimethyl sulfoxide (DMSO) and then diluted with PBS (pH 7.4) to obtain 5 mM KGN working solutions. KGN thermogel was prepared by mixing 100 μl 5 mM KGN solution with 10 ml PLGA-PEG-PLGA gel solution to obtain KGN gel solution containing 50 μM KGN. PLGA-PEG-PLGA gel solution without KGN was used as control.

### *In vitro* KGN Release

The *in vitro* sustained release of KGN from the KGN thermogel was determined by an ultra-micro UV spectrophotometer (Nanodrop 2000). Briefly, 1 mL of 20 wt% thermogel or KGN thermogel solution was placed in a vial of a 16 mm inner diameter for gelation at 37°C. Then 2 mL PBS was added on top of the gel. The supernatant was collected at set time intervals (0.0, 1.0, 3.0, 7.0, 11.0, 15.0, and 20.0 days). Another 2 ml PBS was then replaced. Serial concentrations of KGN in PBS solution were used as standards. The measurement was carried out with a detection wavelength of 277 nm. The amount of released KGN at each time point was calculated as percentage of the total KGN content of a 1 mL 20 wt% thermogel or KGN thermogel solution.

### Isolation and Culture of Chondrocytes

Two-month-old adult New Zealand White rabbits weighing around 1.5 kg were sacrificed for isolation of chondrocytes as previously mentioned (Wang et al., 2016a). Primary chondrocytes were harvested from the cartilage of knees and shoulders. First, the minced cartilage was digested for 6 h in 10 mL of 0.2% w/v collagenase type 2 (Gibco BRL Co. Ltd.) solutions at 37°C. The resultant cell suspension was centrifuged and resuspended in low-glucose DMEM supplemented with 10% FBS (HyCloneTM, Thermo Scientific, Australia) and 1% penicillin and streptomycin. Isolated chondrocytes were cultured in monolayer cultures in a humidified incubator at 37°C, 5% CO_2_, and 21% O_2_. Passage 2 chondrocytes were utilized for subsequent experiment.

### Chondrocytes Treated With IL-1β

Passage 2 adherent rabbit chondrocytes reaching 60–70% confluency was cultured with serum-starved medium (DMEM/F12 supplemented with 1% FBS) for 12 h, and then was treated with IL-1β (10 ng/ml) for 2 h for the following *in vitro* experiments.

### Design of Rabbit Knee OA Model

This study was carried out in accordance with the Guide for the Care and Use of Laboratory Animals of the National Institutes of Health. The protocol was approved by the Committee on the Ethics of Animal Experiments of Xiamen University. The anterior cruciate ligament transection (ACLT) procedure was performed to induce knee OA model as previously reported (Liu et al., 2016). New Zealand white rabbits (*n* = 24, age 5 months, weight 2.5–3.0 kg) were then divided into two groups (sham and ACLT). After anesthesia and routine preparation, 18 rabbits were performed ACLT on the left knees and six rabbits were performed a sham surgery. At 3 weeks after ACLT, rabbits were randomly divided into three groups (six rabbits in each group) for intra-articular injection of saline, thermogel, or KGN thermogel. At 6 weeks after the sham surgery or 3 weeks after the intra-articular injection, the left knees in every group were harvested for histological analysis, and synovial fluid was collected for analysis of interleukin-1 (IL-6) and MMP-13, to evaluate the inflammation of knees.

### *In vitro* Culture of IL-1β Treated Chondrocytes in KGN Thermogel

The cell suspension containing 5.0 × 10^5^ IL-1β treated chondrocytes was mixed with 100.0 μL of thermogel or KGN thermogel solution at 4°C and then transferred into a 24-well plate. The mixed cells–copolymer solution was incubated at 37°C for 15 min for gelation and cell initial attachment. 2.0 mL of fresh DMEM supplemented with 10% (V/V) FBS (HyCloneTM, Thermo Scientific, Australia), and 1% penicillin and streptomycin (Invitrogen, Carlsbad, CA, USA) was added.

For cell proliferation assay and DNA content analysis, the cells-laden thermogel was cultured for 1 week in DMEM. The culture medium was changed every 2 days. The proliferation activity of cells was measured at day 1, 5, and 7 using a Cell Counting Kit-8 assay (CCK-8; Dojindo Laboratories, Kumamoto, Japan) according to the manufacturers' instructions. Briefly, cell culture (*n* = 3) were gently rinsed with PBS and then submerged in a mixed solution of 10.0 μL of CCK-8 reagent with 90.0 μL of fresh medium at 37°C for 2 h. The absorbance readings at 450 nm were observed using a plate reader.

### Biochemical Analysis

To reveal the effect of KGN thermogel on OA chondrocytes, we measured the matrix degradation enzyme (MMP-13), extracellular matrix component [type 2 collagen (COL-2) and glycosaminoglycan (GAG)] secreted from OA chondrocytes. Briefly, the specimens were digested in a pre-prepared papain solution containing 0.5 M EDTA, 0.05 M cysteine hydrochloride, and 1.0 mg/mL papain enzyme (Sigma, St. Louis, MO, USA) at 60°C for 12 h. The aliquots of the sample digestion were used for the measurements of DNA and GAG, as previously reported (Wang et al., 2016a). DNA content was measured using a fluorescence assay. Total glycosaminoglycan (GAG) content was determined using a 1,9-dimethylmethylene blue (DMMB; Sigma, St. Louis, MO, USA) dye-binding assay. The culture medium of IL-1β treated chondrocytes was collected after 3 weeks of culture in KGN thermogel. Supernatant was separated from insoluble residues by centrifugation at 12,000 rpm for 10 min. Rabbit MMP-13 and COL-2 ELISA Kits (Cloud-Clone, Corp., Houston, TX, USA) were used to measure the COL-2 and MMP-13 according to the manufacturer's instructions. GAG, COL-2, and MMP-13 concentrations were normalized to DNA content which was determined fluorometrically using Hoechst staining as previously described (Wang et al., 2016a).

### Gene Expression Analyses

To evaluate the effect of KGN on OA chondrocytes, we measured the expressions of arthritis -related genes and cartilage-related genes. Gene expressions were detected by real-time polymerase chain reaction (RT-PCR) as previously reported (Wang et al., 2016a). At predesignated time points, samples (*n* = 3) were homogenized in Trizol Reagent (Invitrogen, Carlsbad, CA, USA) with a tissue grinder and RNA was extracted according to the manufacturer's instructions. Isolated RNA concentration was determined by an ND-2000 spectrophotometer (Nanodrop Technologies). One microgram of RNA from each sample was reverse transcribed into cDNA using the MMLV Reverse kit (Promega, Madison, WI, USA), and RT-PCR analysis was performed using ABI 7300 real-time PCR system (Applied Biosystems, Foster City, CA, USA) with SYBR Green PCR Master Mix (Toyobo, Osaka, Japan). The relative gene expression was expressed by fold difference which was calculated as 2^ΔΔ*CT*^. The relative expression changes in these target genes were quantified by normalizing their expression to that of housekeeping gene glyceraldehyde-3-phosphate dehydrogenase (GAPDH). PCR primers for: type 1 collagen (COL-1), type 2 collagen (COL-2), aggrecan (AGC), MMP-13, and GAPDH were listed in Table 1.

**Table 1:** Primer sequences used for real-time PCR.

**Gene**	**Forward primers (5^**′**^-3^**′**^)**	**Reverse primers (5^**′**^-3^**′**^)**
*COL-1*	TGGCAAGAACGGAGATGACG	GCACCATCCAAACCACTGAA
*COL-2*	CCACGCTCAAGTCCCTCAAC	AGTCACCGCTCTTCCACTCG
*AGC*	CGTGGTCTGGACAGGTGCTA	GGTTGGGGTAGAGGTAGACG
*MMP-13*	TTGACCACTCCAAGGACCCAG	GAGGATGCAGACGCCAGAAGA
*GAPDH*	CCATCACCATCTTCCAGGAG	GATGATGACCCTTTTGGCTC

*Col-1, type 1 collagen; Col-2, type 2 collagen; AGC, aggrecan; MMP-13, matrix metalloproteinase 13; GAPDH, glyceraldehyde-3-phosphate dehydrogenase*.

### Histological Analysis

After intra-articular injection for 3 weeks, each group of rabbits was euthanatized with overdose of pentobarbital sodium. Distal femur was resected for histological evaluation. No joint infection occurred in all knees. To evaluate the inflammation of knee joints, synovial fluid was collected for analysis of interleukin-6 (IL-6) and MMP-13. After dissection and fixation, the samples were decalcified in 15% EDTA (pH 7.2 in PBS) with 5% paraformaldehyde at 4°C. The decalcified medial condyles were then trimmed, dehydrated in a graded ethanol series and embedded in paraffin. Sections were stained with H&E, TB (positive for proteoglycans) and immunohistochemical (IHC) staining (positive for COL-2). The protocols for detection of COL-2 were described in above sections. The histological sections were blindly reviewed for quantitative evaluation of the cartilage destruction using the Osteoarthritis Research Society International (OARSI) scoring system (Pritzker et al., 2006).

### Statistical Analysis

All data were expressed as means ± standard deviation and represented at least three independent experiments. All data were analyzed using a two-way ANOVA test. *P* < 0.05 were considered significant. When ANOVA results were significant, *post-hoc* analysis was performed via Tukey's multiple comparison test. All analyses were carried out using GraphPad Prism version 6.0 for Windows (GraphPad Software, San Diego, CA).

## Results

### *In vitro* Assessments of KGN Release

**Figure 1:**
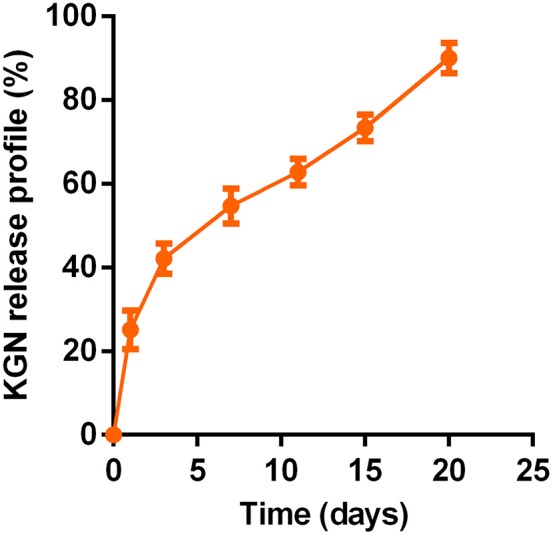
*In vitro* sustained KGN release from KGN thermogel (20.0 wt%) in PBS (pH 7.4) at 37°C (*n* = 3).

The PLGA–PEG–PLGA copolymer in PBS (20 wt%) with or without KGN showed stable gelation at 37°C. The sustainably release KGN *in vitro* from the KGN thermogel determined whether the functionalized thermogel can produce sufficiently chondrogenic effects (commonly used concentration is 100 nM−10 μM *in vitro* and 10 μM−100 μM *in vivo*). The KGN concentration in the 20 wt% polymer solution was about 50 μM. Therefore, the KGN thermogel showed an abrupt release (20%) of KGN at day 1 (10 μM), followed by a sustained release (about 3% per day) of KGN per day (1.5 μM /day), as shown in Figure 1.

### Cell Viability and Proliferation

**Figure 2:**
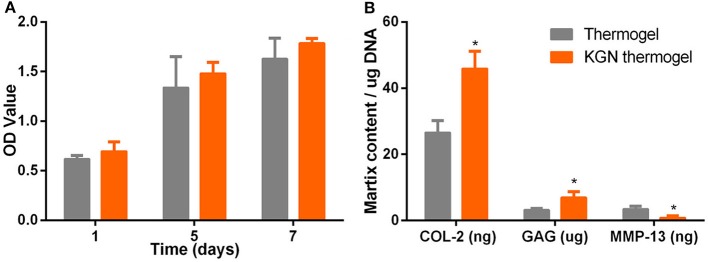
*In vitro* cell proliferation and matrix component production. **(A)** CCK-8 assay showed that the proliferation of IL-1β treated chondrocytes in thermogel or KGN thermogel increased over time **(B)** GAG content was measured by DMMB assay, COL-2, and MMP-13 were determined by ELISA after IL-1β treated chondrocytes were cultured in thermogel or KGN thermogel for 2 weeks (*n* = 3, *P < 0.05).

After culture in growth medium for 72 h, CCK assay showed that OA chondrocytes proliferated in both PLGA-PEG-PLGA thermogel and KGN thermogel showed an increased proliferation during 7 days of *in vitro* culture (Figure 2A). However, the number of OA chondrocytes in thermogel and KGN thermogel on Day 7 did not differ significantly, compared to that on Day 1 (*p* > 0.05). Notably, the number of MSCs in KGN thermogel slightly surpassed that in the thermogel.

### Extracellular Matrix Related Component Production

GAG and COL-2 content were detected to quantify cartilaginous matrix production by OA chondrocytes. Increased amount of GAG and COL-2 from OA chondrocytes were found after culture in thermogel or KGN thermogel for 14 days (Figure 2B). Significantly higher amount of GAG and COL-2 was detected in KGN thermogel than that in thermogel group (*p* < 0.05). On the other hand, increased secretion of MMP-13 was only found in thermogel, whereas minimal increase of MMP-13 was detected in KGN thermogel. These results showed that KGN enhanced the production of COL-2 and GAG and inhibited the production of MMP-13 from OA chondrocytes, suggesting that KGN played an anti-arthritic role in OA chondrocyte-laden thermogel.

### Cartilage-Specific Gene Expression Analyses

**Figure 3:**
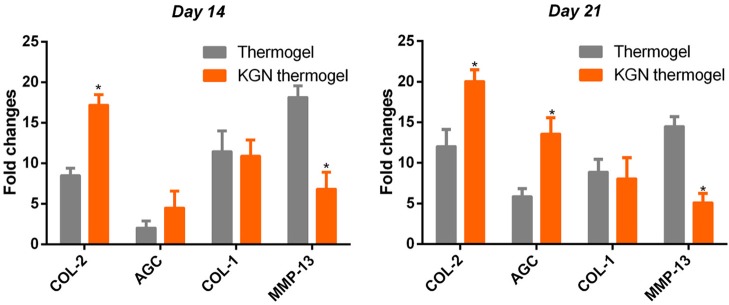
Expression of cartilage-related genes of COL-2 and AGC, COL-1, and MMP-13 of OA chondrocytes within thermogel with or without KGN (*n* = 3; *p < 0.05).

To compare the chondro-protective capacity of KGN thermogel, we measured gene expressions of COL-1, COL-2, AGC, and MMP-13. Significant differences of gene expression were found between thermogel group and KGN thermogel group at both 14 and 21 days of *in vitro* culture (Figure 3). Greater upregulation of hyaline-cartilage specific genes COL-2 and AGC were detected in KGN thermogel system that that in thermogel with no KGN (*p* < 0.05). However, the expression of fibrocartilage-related marker COL-1 was similar between the two groups. In addition, the MMP-13 expression was reduced in KGN thermogel group suggesting KGN could inhibit matrix degradation.

### *In vivo* Cartilage Regeneration

**Figure 4:**
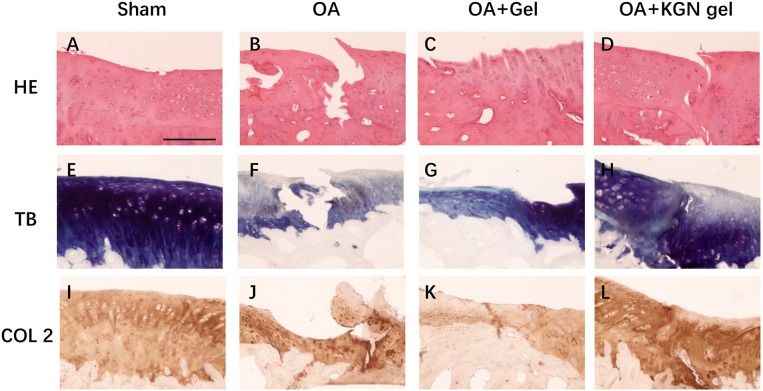
Histological staining of OA knee treated with saline (OA), thermogel (OA + Gel), and KGN thermogel (OA + KGN gel) at 6 weeks after ACLT surgery. **(A–D)** H&E, **(E–H)** toluidine blue (TB), and **(I–L)** immunohistochemistry (IHC) COL-2 staining.

The histological staining showed that the OA model was successfully established in rabbit knees at 3 weeks after ACLT. Obvious OA changes of cartilage occurred at 6 weeks after ACLT in OA group, OA + Gel group, and OA + KGN gel group, with cartilage fibrillation, surface erosion, fissure, denudation, and deformation (Figure 4). Slight cartilage degeneration was observed in sham group with chondrocyte hypertrophy and clustering. Importantly, we noted a distinct chondroprotective effect of KGN thermogel in OA + KGN gel group which showed shallow vertical fissures of the superficial cartilage, localized proteoglycan depletion, and partial COL-2 loss in limited zones of the cartilage. In comparison, both OA and OA+ thermogel groups showed severe OA changes, with significant cartilage denudation and deformation, as well as marked proteoglycan and COL-2 depletion. These data suggested that IA injection of KGN showed an anti-inflammatory effect and promoted cartilage regeneration, as compared to IA injection of saline or thermogel.

**Figure 5:**
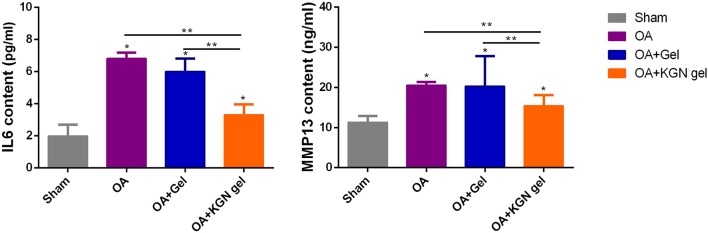
ELISA method detecting IL-6 and MMP-13 content in synovial fluid of all groups at 6 weeks (*n* = 8, *p < 0.05, **p < 0.01).

OA knees treated with KGN thermogel injection demonstrated delayed cartilage degeneration and low level of articular inflammation, as indicated by IL-6 and MMP-13 content. In contrast, the IL-6 and MMP-13 levels were significantly higher in OA and OA + Gel groups (*p* < 0.01; Figure 5).

**Figure 6:**
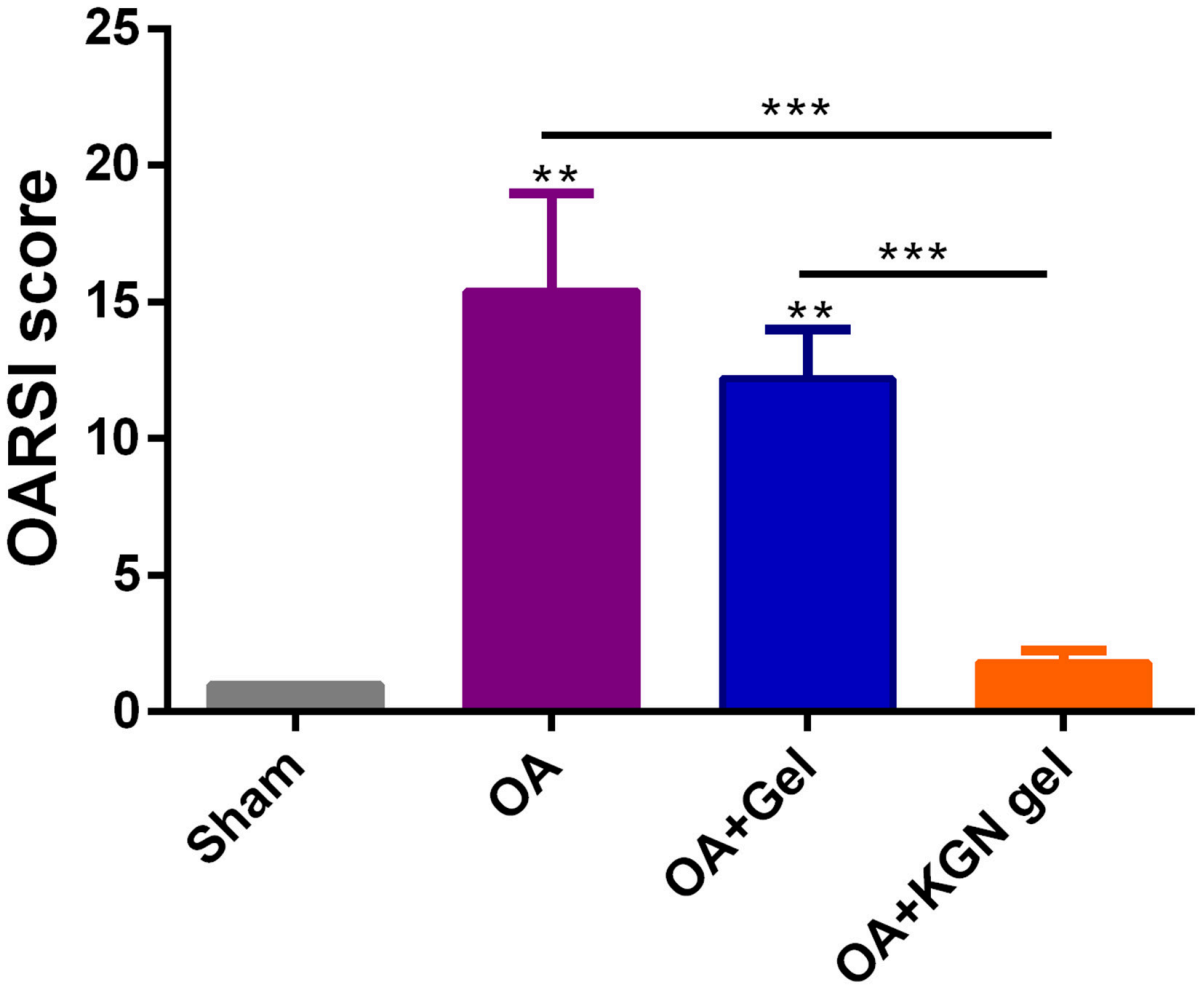
OARSI scores were significantly lower in Sham group and OA + KGN gel group that those of OA and OA + Gel groups (**p < 0.01, ***p < 0.001).

The OARSI scores grade histopathology of OA based on extent of joint involvement and the depth of lesion (Pritzker et al., 2006). As shown in Figure 6, OARSI scores were significantly lower in sham group and OA + KGN group that those of OA and OA + Gel groups (*p* < 0.001). The OA group treated with saline IA injection displayed progressive cartilage degeneration with extensive and deep involvement of cartilage. The OARSI scores revealed no significant differences between OA gel group and OA group (*p* > 0.05). These data suggested ongoing arthritis and cartilage destruction in OA group and OA + Gel group, and subsided arthritis and regenerated cartilage in arthritic knees treated with KGN thermogel.

## Discussion

OA is characterized by extracellular matrix (ECM) degradation and cell loss. The main components of ECM in cartilage are proteoglycans and collagens, and the loss of AGC and COL-2 in ECM leads to cartilage degradation. Matrix metalloproteinases (MMPs), for example, MMP-13, have been shown to directly cleave collagens and aggrecan (Zhao et al., 2014).

The close articular cavity allows the possibility of IA drug administration for OA patients. IA injection of drugs will avoid or minimize side effects of systematically administered drugs, such as toxicity and gastrointestinal complications (He et al., 2017). However, the low retention time limits the application of IA injection. Hydrogels are physically or chemically cross-linked three-dimensional polymers swollen in water that allows the controlled release of bioactive agents (Park et al., 2009) and possesses the capacity of maintaining chondrocyte phenotype (Chung and Burdick, 2008; Spiller et al., 2011; Liu et al., 2018). Among various hydrogels, the thermo-sensitive hydrogel from PLGA–PEG–PLGA copolymer has been especially attractive, as their spontaneous gelation under physiological conditions presents some advantages: minimal invasive wounds, efficiency of cell encapsulation and controlled release of bioactive molecules (Zhang et al., 2014, 2015; Li et al., 2016).

In the current work, the cartilage-protective molecule KGN was incorporated into the PLGA–PEG–PLGA thermogel with the capacity of sustained release of KGN for as long as 3 weeks. We used IL-1β treated rabbit chondrocytes to investigate the effect of KGN on OA chondrocytes. *In vitro* experiment showed that KGN thermogel was compatible with cell survival, however with no capacity to promote proliferation of OA chondrocytes. In addition, when cultured in KGN thermogel, OA chondrocytes produced higher amount of GAG and COL-2 than those in thermogel without addition of KGN. These data are consistent with others which showed that KGN promoted cartilage regeneration and induced chondrogenic differentiation of mesenchymal stem cells (Kang et al., 2014, 2016; Wang et al., 2016b; Fan et al., 2018; Han et al., 2019). In addition, the level of MMP-13 produced by OA chondrocytes in KGN thermogel was much less compared to that in thermogel. This is consistent to the increased amount of COL-2 and GAG, which can be directly degraded by MMP-13. Therefore, KGN thermogel system have showed biocompatibility and cartilage-protectiveness. The gene analysis was in line with the above ELISA results, as KGN thermogel group enhanced expression of hyaline-cartilage specific genes COL-2 and AGC, and inhibited the expression of MMP-13. This result is again supported by other researchers (Wang et al., 2015; He et al., 2017; Patel et al., 2019).

To confirmed the feasibility of using KGN thermogel for the treatment of OA, we established knee OA model using ACLT procedure. The OA knees treated with KGN thermogel yielded significantly greater cartilage regeneration and less joint inflammation, as suggested by histological staining, OARSI score and synovial fluid analysis of inflammatory cytokines. These *in vivo* data strongly indicated that KGN played an anti-arthritic and chondro-protective role in arthritis.

Some limitations remain in the present study. Firstly, the current study is only a preliminary study for the effect of KGN on the early stage OA. However, more time points for IA injection at different OA stages will add more clarification for the effect of KGN on OA treatment. Secondly, the potential molecular mechanism regarding the effect of KGN on OA was not revealed in the current study. Further studies about KGN in anti-inflammation or redifferentiation of OA chondrocytes should be needed in the future.

In summary, we have shown that KGN thermogel can release KGN both *in vitro* and *in vitro*. IA injection of KGN thermogel can enhance cartilage regeneration and inhibit joint inflammation in OA knees in a rabbit model. KGN thermogel protects the cartilage by promoting OA chondrocytes to produce COL-2 and GAG, while reducing the secretion of MMP-13.

## Data Availability Statement

The raw data supporting the conclusions of this manuscript will be made available by the authors, without undue reservation, to any qualified researcher.

## Ethics Statement

The animal study was reviewed and approved by Committee on the Ethics of Animal Experiments of the University of Xiamen.

## Author Contributions

S-JW and J-ZQ: conception and design, collection and assembly of data, analysis and interpretation of data, and drafting of the manuscript. T-EZ: assistance in analysis and interpretation of data. CX and S-JW: conception and design, critical revision of the manuscript, and final approval of the manuscript.

### Conflict of Interest

The authors declare that the research was conducted in the absence of any commercial or financial relationships that could be construed as a potential conflict of interest.
